# Impact of Electrode Position on the Elicitation of Sodium Spikes in Retinal Bipolar Cells

**DOI:** 10.1038/s41598-017-17603-8

**Published:** 2017-12-14

**Authors:** Frank Rattay, Hassan Bassereh, Andreas Fellner

**Affiliations:** Institute of Analysis and Scientific Computing, Vienna University of Technology, Vienna, Austria

## Abstract

Bipolar cells of the magnocellular pathway in the primate retina can generate action potentials because they have an axonal segment with high sodium channel density, comparable to the sodium channel band in retinal ganglion cells or pyramidal cells. The similarity between the non-human primate and the human retina is of interest for the research on retinal implants for the blind, and especially, the conditions to elicit sodium spikes in bipolar cells using extracellular stimulation. A comparison of excitation characteristics of three model neurons, a bipolar cell, a retinal ganglion cell, and a cortical pyramidal cell, demonstrates the similarities and differences regarding stimulation with microelectrodes. Moving a microelectrode parallel to the axon of a neuron commonly allows to generate spikes for every position – and this rule holds both for cathodic and anodic pulses. However, for the simulated bipolar cell anodic pulses cannot generate sodium spikes directly. Further, there is only a small region for electrode placing where extracellular cathodic stimulation causes direct spike initiation in the sodium channel band. For all other positions, a sodium spike can only be generated by antidromic current flow originating from strongly depolarized terminals.

## Introduction

Artificial spikes of retinal ganglion cells (GC) generated by inner eye prostheses lead to visual perceptions. These spikes are caused directly by stimulation of GC axons or indirectly via synapses from stimulated bipolar cells (BC), the interneurons between photoreceptors and GCs^1–4^. Stimulation with electrodes close to BCs allows a patient to use the optic features of his eye while it is assumed that artifacts resulting from co-stimulation of GC axons originating in distant regions can be avoided^5–7^. Even though BC stimulation seems to be closer to the eye’s natural signaling pathway, many features of BC responses to electrical stimulation are not clear. It was a well excepted doctrine that GCs are spiking cells whereas signaling of all other retinal neurons is performed via graded potentials only. However, recently sodium and calcium spikes were recorded in fish and mammalian BCs^8–12^. From the neural engineering point of view, experiments on non-human primates are of specific interest as their BCs are similar to that of humans^9^.

Bipolar cells of the magnocellular pathway in the primate retina are involved in motion and flicker detection where high temporal resolution is needed. Their ability to generate sodium spikes supports the quick signal transport from photoreceptors to GCs as, compared to graded potentials, a spike causes stronger synaptic activity at the BC terminal^11^. This first computer simulation study on spiking in BCs evoked by extracellular stimulation deals with sodium spikes because this physiological characteristic is shared with other neurons.

**Figure 1:**
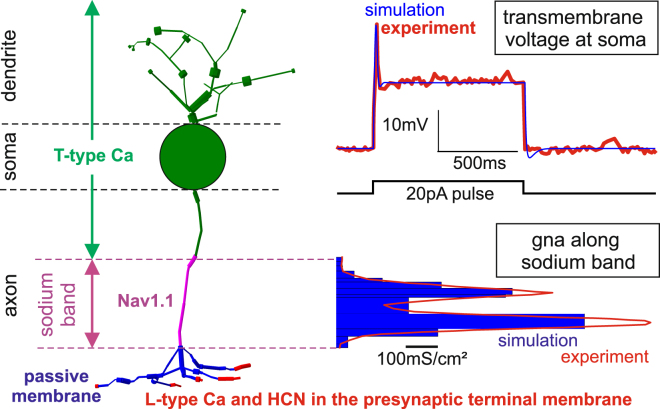
3D model of a DB4 BC consisting of a spherical soma (d = 10.3 µm) and 116 cylindrical compartments (left), the distribution of the sodium ion channels which amplify excitation (bottom right), and the recorded and simulated sodium spike as a response to intracellular soma stimulation (top right). The colors in the left panel symbolize the main types of the transmembrane currents along the cell axis. A peak conductance value close to g_Na_ ≈ 1000 mS/cm² for voltage sensitive sodium channel type Nav1.1 dominates the role of sodium ions when generating spikes. Calcium spikes are not generated in this cell type, as the large membrane surface containing calcium ion channels (green) cannot compensate the low calcium conductance value g_Ca_ between 1 and 2 mS/cm². The values of the blue g_Na_ histogram were used as individual conductance parameters for the 9 sodium band compartments. Cell geometry and experimental data from ref.^9^.

The sodium band, an axon segment with high density of voltage sensitive sodium channels that is often called axon initial segment (AIS), is the preferred site of spike initiation in almost all types of neurons^13–15^. Depending on the type of neurons, the sodium band differs in channel density, length and diameter, and its distance to the soma (Table 1). The sodium channel density of the sodium band, quantified by the maximum sodium ion conductance value g_Na_ in Table 1, is assumed to be distributed with more constant density in a GC or a pyramidal cell (PC)^14,15^. In contrast the simulated BC, where most data are based on ref.^9^, the sodium channels have a bimodal density distribution with a mean value of around 350 mS/cm² (Fig. 1, bottom right). The shape of the simulated transmembrane voltage fits the experimentally determined data (Fig. 1, upper right graph). Note the small spike amplitude of only ~30 mV (recorded at the soma) as response to a 20 pA current injection into the soma. More details about the investigated (diffuse bipolar) DB4 cell of macaque can be found in ref.^9^.

**Table 1:** Morphometric and sodium channel density data of the three investigated model neurons.

	Bipolar Cell	Ganglion Cell	Pyramidal Cell
Dendrite g_Na_ (mS/cm²)	0	25	8
Soma g_Na_ (mS/cm²)	0	80	8
Axon g_Na_ (mS/cm²)	0	70	300
Sodium band g_Na_ (mS/cm²)	350	400	420
Soma diameter (µm)	10.3	20	20
Sodium band diameter (µm)	0.5	2	1.22
Sodium band length (µm)	26	40	50
Distance between sodium band and soma (µm)	4.7	40	10
Main reference	Puthussery *et al*.^9^	Rattay 2014^18^	Rattay *et al*.^17^
Additional references		Jeng *et al*.^35^, Sheasby *et al*.^39^	Hu *et al*.^15^, Mainen & Sejnowski^13^

Bipolar retinal cells have an extremely short axon and consequently the undersized sodium band limits the number of Na channels. Thus, a limited signal amplification is causing only a spikelet (Fig. 1) rather than an action potential where an amplitude greater than 100 mV would be expected. This is not a fundamental physiological problem as the cell operates both via action potentials and via graded potentials. However, sending a spike to the synaptic terminals results in a larger amount of released neurotransmitter at the synaptic connections to GCs which has direct consequences for visual perception, e.g. by less jitter in the neural pattern^11^.

This computer study aims different questions regarding the behavior of BCs to extracellular stimuli. While some of the questions are directly related to the biophysical properties of the BC such as ion channel distribution, other questions are strongly connected to the geometric setup of the electrode in relation to the target cell. The position of the electrode determines de- and hyperpolarizing effects along the neuron. Of special interest in this study are the electrical effects at the ends of the neurites. While in large structures their impact (far from the stimulating electrodes) can often be neglected, on a small structure like a BC these effects may play a dominant role in the signal processing of the cell. A multi-compartment model is a useful tool to answer arising biophysical and geometric questions and to analyze the driving forces from intracellular versus extracellular stimulation.

Plenty of our electrophysiological knowledge is based on experiments where the transmembrane voltage V_m_ of a cell is directly influenced by an electrical stimulus in order to trigger the active cell mechanisms which are based on voltage sensitive ion channels. The transmembrane voltage is the difference between intra- and extracellular potentials (V_m_ = V_i_ − V_e_). At rest, the negative resting voltage V_r_ is only determined by the negative intracellular potential V_i._ A more positive membrane voltage (>V_r_) is referred as depolarization while a more negative one is referred as hyperpolarization. One way to influence the membrane voltage *in vitro* is the intracellular stimulation where positive current pulses are intracellularly applied into the soma. The injected current is distributed via intracellular current flow along the whole neuron causing a rise of intracellular potential which leads to a depolarization along the whole cell.

**Figure 2:**
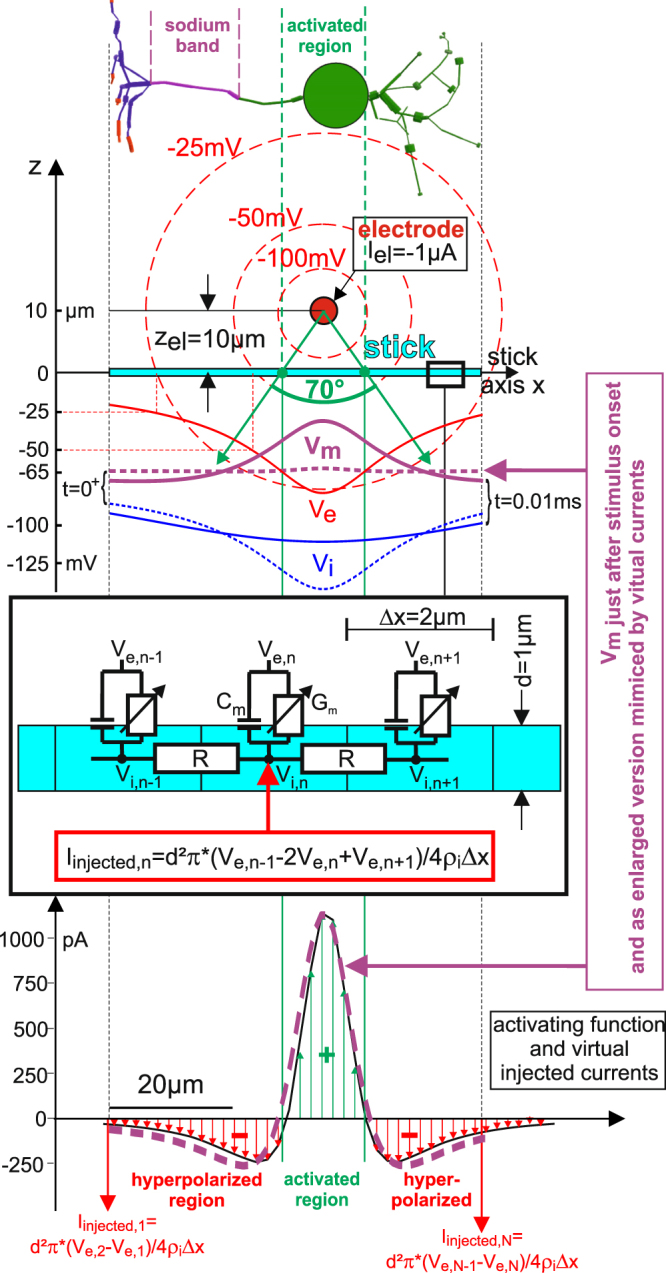
Injected currents from virtual intracellular electrodes replace extracellular point source stimulation in a stick model of the BC. Positions of point source and stick define an angle of 70 degrees which separates the depolarizing and hyperpolarizing driving forces (top and bottom) under the assumption of a large homogeneous medium and distant ground electrode. This 70° angle is independent of intra- and extracellular resistivities or membrane parameters, see^27,40^ for details. While the extracellular potential V_e_ (red) is assumed to be time invariant during the stimulus pulse, the intracellular potential V_i_ (blue) balances out during the stimulus along the cell. These intracellular balancing forces have direct effects on the membrane voltage V_m_ (purple). Broken lines indicate V_i_ and V_m_ just after the stimulus onset (t = 0^+^), full lines at t = 0.01 ms. Because the pulse is cathodic, depolarization will take place right below the electrode which is already visible in V_m_ at t = 0.01 ms). The electrical circuits (center) define a main equation of the Hodgkin-Huxley type for each compartment in order to simulate the neural response to a stimulus pulse from the point source. However, neither membrane capacitance C_m_ nor voltage dependent membrane conductance G_m_ are needed to find the pulse amplitudes of the virtual electrodes for the central compartments (formula at center) and end-compartments (formulas at bottom). Irregularities in the activating function (bottom) are consequences of low spatial discretization. Moving the electrode parallel to the x-axis does not change the characteristic shape of the central (green) region, but will cut or respectively extend more or less of the red wings at the ends. As the sum of the green and red currents has to be 0, the last compartment N will respond to a point source shift to the right side with an increase of the last red arrow, whose length is equal to the sum of all arrows that are cut away of the right side of the (theoretically infinite) long stick. Note, the shown point source position is not favorable to elicit an AP in the sodium band as the sodium channels are outside of the green region; furthermore, the low Ca-channel density in the soma region may not amplify the membrane voltage enough. Application of a positive pulse has a better chance as the red region on the left wing which overlays the sodium band would then be depolarizing. However, to predict when and where APs are elicited a better model is required that includes (i) the cell geometry (differing diameters, bifurcations, 3D pathways of neurites) and (ii) the kinetics and densities of all involved ion channel types.

However, for extracellular stimulation, e.g. as needed in clinical applications, the extracellular potential is given by the applied electric field. Depending on the electrode position related to a target neuron and the electrical properties of the cell, an extracellular stimulus causes depolarized regions and hyperpolarized regions of different strength along the neuron at the same time. The de- and hyperpolarizing forces are independent of any active mechanisms of the cell, they are just caused by the passive behavior of a cell inside an electric field. When a stimulating square pulse establishes a time-invariant extracellular potential along the surface of the cell, the electric forces inside the cell tries to balance out the intracellular potential which is effected by the stimulus, too. This leads to a smoother intracellular potential (Fig. 2, blue curves for V_i_ at t = 0^+^ and t = 0.01 ms) compared to the time invariant extracellular potential (red curve). As no active mechanisms are present at the early beginning of the stimulus, the average membrane voltage along the whole cell is still on its resting level Vr as no charged ions entered or left the cell, The difference between intra- and extracellular potential results in depolarized and hyperpolarized regions. For a cathodic pulse, depolarization will occur below the electrode while the flanks are hyperpolarized. For anodic pulses it is vice versa.

Before presenting specific results of the computer simulation, a closer look at extracellular stimulation and its direct passive effects on a cell is taken by outlining the concept of the activating function^16^. Any extracellular stimulation case can be imitated by an equivalent intracellular stimulation configuration, where in every compartment a virtual intracellular electrode is placed. The virtual electrode injects a current equivalent to the capacitive current through the membrane that can flow laterally between compartments. Since the incoming capacitive current and the lateral current are inversely dependent on the membrane properties (conductance and capacitance), the effect of these membrane properties cancels so the virtual current depends only on the change in the external voltage field over space and time, cell geometry, and internal resistivity. A virtual intracellularly applied electrode current that substitutes the effect of the extracellular electrode can be calculated by the extracellular potentials V_e_ of the three compartments involved, the intracellular resistivity ρ_i_, the compartment diameter d, and the compartment length Δx. This is demonstrated in the formula for the n-th compartment in Fig. 2, center. The virtual currents are described by the activating function^17^.

Important conclusions of the activating function concept are: (i) the sum of all virtual electrode currents is zero at any time of stimulation (ii) regions with positive virtual electrodes are candidates for spike initiation, (iii) regions with negative virtual electrodes are candidates for hyperpolarization. Rule (i) implies the (total) driving forces that create hyperpolarization are of equal strength as those for depolarization along the neuron because the total current flux into the cell has to be equal to the flux out of the cell. Consequently, the sum of all virtually injected currents is zero which is seen also from the formulas in Fig. 2.

$$\begin{array}{ccc}0 & = & {d}^{2}\pi /4{\rho }_{i}{\rm{\Delta }}x(-Ve,1+Ve,2\\  &  & +Ve,1-2Ve,2+Ve,3\\  &  & +Ve,2-2Ve,3+Ve,4\\  &  & +Ve,3-2Ve,4+Ve,5+\ldots )\end{array}$$

Here, the line number corresponds with the compartment number. All Ve values cancel each other out and the sum of the virtual currents becomes zero.

Some important consequences from the activating function concept are documented in the example where the BC geometry is replaced by a single cylinder (stick model, Fig. 2). The activating function is about 4 times larger in the center (near electrode) than at its lateral wings. For cathodic stimulation the green currents are depolarizing, for anodic stimulation the red ones (lower part, Fig. 2). This directly implies lower stimulus thresholds for extracellular cathodic pulses compared to anodic pulses. Next, for short neurons such as the BC, the ends may cause surprising effects as rather large virtual electrode currents may appear there. As rule (i) states that the sum of all de- and hyperpolarizing currents is zero, high virtual currents on the ends must balance out the large currents near the electrode. Furthermore, a low threshold current for the extracellular stimulating electrode is predictable for positions causing high positive virtual electrode currents in a region with high sodium channel density (sodium band). Because of the short sodium band in BCs (Fig. 1), only a limited range of electrode positions should be expected for direct sodium spike initiation.

In the following computer simulation study electrophysiological properties of a representative BC, a GC, and a cortical PC are compared to emphasize the particular features of a BC under electrical stimulation. For this reason, the model cells are analyzed regarding their excitation with respect to different kinds of stimulation and changing electrode positions. In order to find basic results, the three model cells are assumed to be within a large homogeneous medium of constant conductance, the stimulating microelectrode is simulated as a point source generating monophasic square pulses, and the ground electrode is assumed to be far away.

## Results

### The sodium band is the most excitable part of the BC

**Figure 3:**
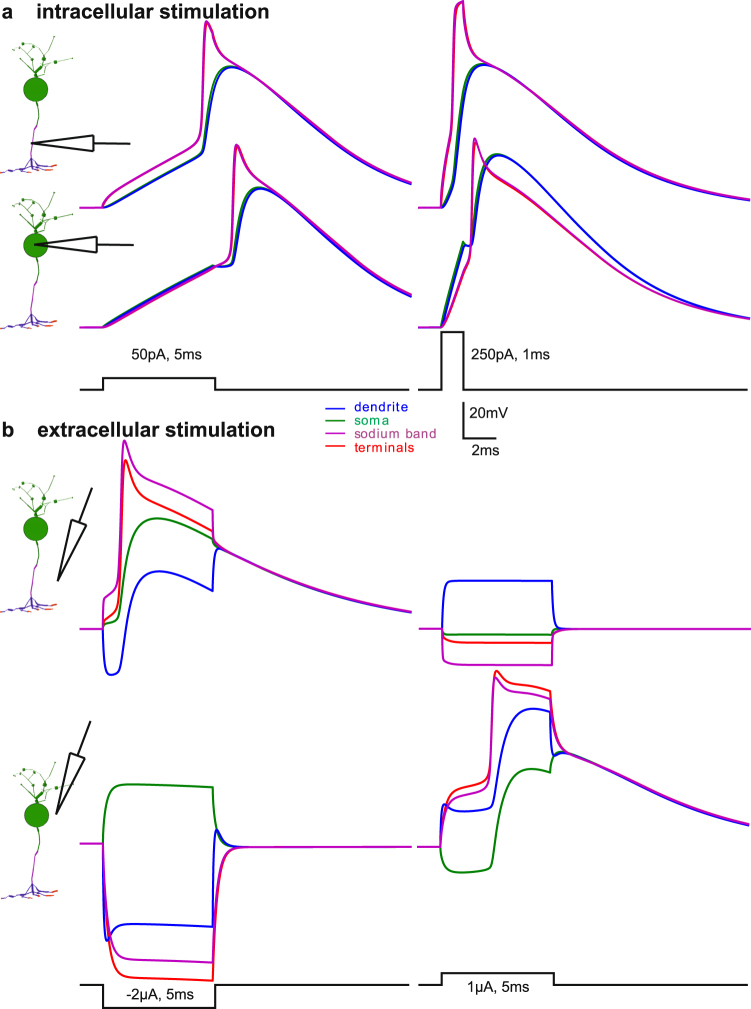
Transmembrane voltages for intracellular vs. extracellular BC stimulation. An intracellularly elicited spike in the sodium band propagates into soma with short delay (**a**, upper graphs) and causes earlier spikes compared to somatic current injection (**a**, lower graphs). Because of the close distance, the curves for sodium band and terminal are coincident, same for dendrites and soma. Short (1 ms) pulses of the same charge cause the same excitation characteristics but little higher AP peaks than for longer pulses. The difference in the AP magnitudes is caused by the slow potassium channels in the soma. (**b**) Extracellular stimulation causes hyperpolarized and depolarized regions during pulse application which quickly result in a common transmembrane voltage for all segments once the pulse is over. Electrode distance to cell axis 10 µm. Resting membrane voltage is −70 mV.

This is demonstrated by the timing of the spike in the sodium band compared to other positions of the cell. No matter if an anodic current pulse is injected into the sodium band or into the soma, in both cases the spike is initiated first in the sodium band (Fig. 3a). The small cell size with a maximum extension of 61 µm in axial direction ensures a fast conductance of the spike to the terminals resulting in coincident voltage curves of sodium band and terminals. As a consequence of the small ratio (surface of sodium-band)/(surface of soma), the signal appears with a reduced amplitude and a delayed peak at the soma and dendrite. In comparison with a GC or a PC (Table 1), the rather small number of sodium channels in the BC cannot charge the large capacitance of the soma with enough axonal current to generate a somatic spike with the same amplitude.

Whereas anodic intracellular stimulation per se creates no hyperpolarization along the whole cell (Fig. 3a), cathodic extracellular stimulation generates both depolarized and hyperpolarized regions (Fig. 3b, left). Changing the electrode polarity from cathodic (Fig. 3b, left) to anodic (Fig. 3b, right) causes the regions which were originally hyperpolarized to become depolarized (and vice versa). The hyperpolarized dendrites (left upper part in Fig. 3b) becomes the most depolarized BC segments when stimulus pulse polarity is changed (right upper part in Fig. 3b). However, for this anodic case the dendritic calcium channel density is too low to elicit a Ca-spike. Interestingly, the small shift of the electrode position from sodium band to the soma needs a change of stimulus pulse polarity to elicit a spike (compare Fig. 3b upper left to lower right).

Another important point is how the spike is generated in the sodium band. Cathodic stimulation above the sodium band initiates the spike within the sodium band. This is demonstrated from the first moment of stimulation where the purple curve for the sodium band is always the most depolarized one (upper case in Fig. 3b). However, for anodic stimulation the terminal segments represented by the red curves are mostly depolarized (lower right case in Fig. 3b) which then activate the sodium band by intracellular current flow.

### Bipolar cells versus ganglion and pyramidal cells

Although BCs and GCs are both retinal cells with small extensions orthogonal to the retina surface, there are several essential differences: GCs usually have a larger soma diameter, larger dendrites, and a very long axon with a longer sodium band. Summarized, the relationship of GCs to standard neurons, e.g. PCs, is much closer than to BCs concerning (i) long axons, (ii) sodium band properties, and (iii) response characteristics to electrical stimulation.

**Figure 4:**
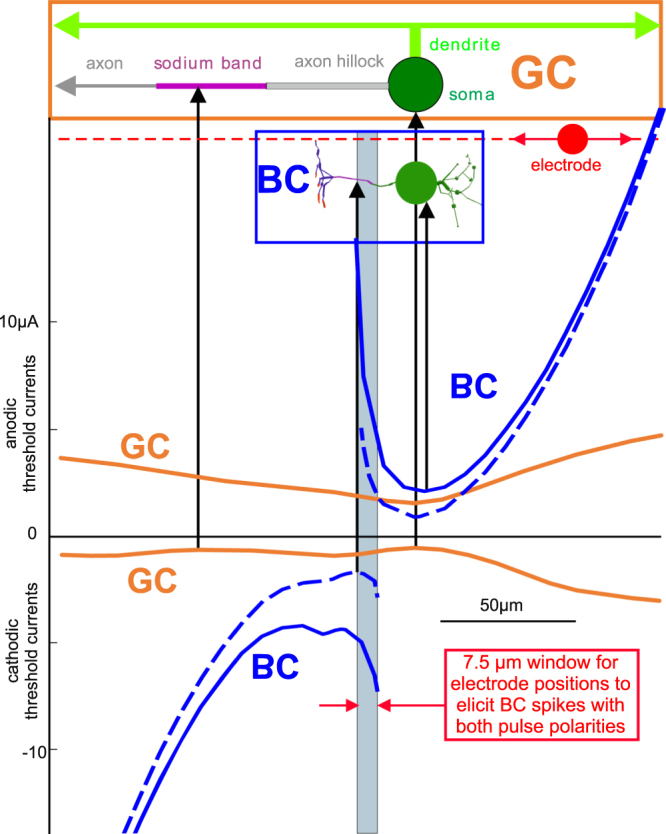
Anodic and cathodic threshold currents for the bipolar and the ganglion cell when an extracellular stimulating electrode is placed at a distance of 20 µm (full lines) and 10 µm (broken lines) from the axon. Compared to the BC, the GC has lower thresholds with two minima for cathodic pulses (marked by arrows), one at the closest position to the soma, the other one similar to the BC above the sodium band. Note, while for the GC both polarities will lead to excitation at any electrode position, the BC can be stimulated only with one polarity for most electrode positions. Only for a small area above the initial axon segment (marked in light blue) both polarities will cause excitation. Note, in reality the orientation of the GC’s axon and dendrites is parallel to the retina surface and thus orthogonal to the BC orientation, but here, for comparison, BC is rotated to be parallel to the shifting axis of the electrode. Pulse duration is 10 ms.

For a comparison of BC vs. GC, the GC geometry is reduced to a case similar to ref.^18^. The model cell has a long straight axon and the dendrite tree of a real cell is substituted by a short dendrite having two branches, both 300 µm long and parallel to the axon (upper part of Fig. 4).

Comparing intracellular threshold currents for somatic stimulation of the different model cells in Table 2, increasing currents in the order BC, GC, and finally PC can be observed. The lower threshold of the BC can be explained by the smaller BC soma diameter (d = 10.3 µm) in combination with only a few dendrites. PC and GC have the same soma diameter of 20 µm, but the PC has the larger dendritic tree with many branches close to the soma which increases the required current for spike initiation. For current injection directly into the sodium band, the situation is slightly different between PC and GC. Here, the PC needs less stimulus intensity than the GC because of the presence of low threshold voltage channels Nav1.6 in the PC axon^15^. But because of its small size, the BC also has the lowest value for sodium band stimulation.

**Table 2:** Intracellular threshold currents in pA.

Pulse duration & Injection site	1ms soma	1ms sodium band	10 ms soma	10 ms sodium band
BC	221	151	29	27
GC	758	639	127	127
PC	1000 (4300)	220 (220)	280 (320)	120 (150)

The PC values are for a 1D cell model and (in brackets) for a traced 3D model. The large number of basal dendrites originating at the soma is not covered enough by the 1D PC model which is causing low threshold currents especially for short pulses compared to the more realistic 3D model^17^.

Keeping the low threshold values of the BC for intracellular stimulation in mind, it might be surprising that for extracellular cathodic stimulation the BC thresholds are essentially higher than that of GC (full lines in the lower part of Fig. 4 for the 20 µm electrode distance). A confirmation of this phenomenon is supported by the activating function concept. Figure 2 shows the virtual electrodes for the BC stick model for a point source distance of 10 µm. Increasing this distance to 20 µm doubles the size of the central region where the virtual intracellular currents are positive for cathodic stimulation, resulting in essentially larger negative side effects at the stick ends. Using a long stick representing the GC would essentially reduce the side effects from the ends. Here, the virtual current model can be simplified to a tri-pole where intracellular current flow reduces the stimulating impact depending on the distances between the poles and the intracellular resistance (number of involved compartments times R according to the electrical circuit diagram of Fig. 2). The intracellular resistance is low for the BC on the side that includes the soma (in contrast the case where the electrode is above the GC sodium band). Both effects reduce the stimulating efficiency for a short cell.

**Figure 5:**
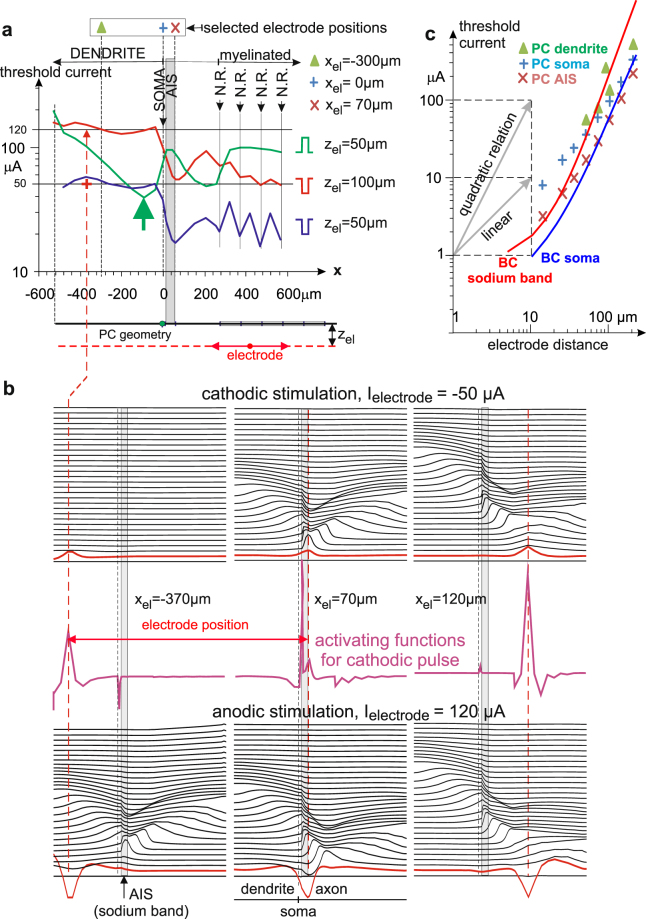
Anodic and cathodic threshold currents for the one-dimensional pyramidal cell when an extracellular stimulating electrode is moved along the axon (**a**), transmembrane voltage profiles along the cell (**b**) and radial current distance relationship for selected electrode positions (**c**). The lowest PC thresholds for cathodic pulses are at the distal end of the sodium band (marked as AIS, gray area) and at the nodes of Ranvier (N.R.) in the myelinated axon. The thresholds for anodal stimulation are essentially larger than for cathodic pulses (green vs. blue lines in (**a**)). A special exception is marked by the large green arrow. At this electrode position, the depolarizing right wing of the activation function triggers an AP in the AIS region when stimulated with an anodic pulse. A similar situation is seen in the lowest central graph where the spike is initiated in some positive x distance to the electrode position. Note, the first transmembrane voltage responses (red line in graphs of (**b**) are smoothed versions of the respective activating function (which has to be inverted for the anodic stimulation cases). Some further examples of AP generations for both stimulus pulse polarities are shown in (**b**) with vertically time shifted voltage profiles; shifted lines correspond to 100 µs intervals. Graph c compares current distance relations for different electrode positions for extracellular stimulation. Results for the BC are shown by lines, the ones for the PC are indicated with markers, different electrode positions are named inside the graph. The depicted cases are for cathodic stimulation with exception of BC soma (blue line) which is, in agreement with Fig. 4, only possible for anodic stimulation. The use of logarithmic scaling demonstrates the trend of changes from linear to quadratic current – distance relations. Pulse duration was 100 µs for PC but 10 ms for BC. In (c) it seems that BC is more sensitive than PC. However, strength duration curve for the investigated PC indicate a sensitivity shift by a factor of about 10 in direction of lower thresholds when pulse duration is increased to 10 ms^17^.

In BCs, evoking spikes with external stimuli of both polarities is possible only in a small region of about 7.5 µm length, but such a restriction is not seen for the GC and the PC (Figs 4 and 5). In general, in most cases anodic extracellular stimulation requires higher stimulation strength than cathodic stimulation (see for example GC in Fig. 4)^16,19^. But in contrast to this usual cell behavior, the BC needs the smallest electrode currents for anodic stimulation, not for cathodic one (Fig. 4). Another exception is documented for the PC, however for a small region where the electrode is above the dendrite and close to the soma (green arrow in Fig. 5a). Some explanations for these surprising phenomena are given in the next subsection and in the discussion which underlines the impact of intracellular current flow between the compartments on spike initiation site.

### Spike initiation site

For the GC different sites of spike initiation were determined. All parts of the modelled GC have sodium channels with significant densities. Therefore, every part should be able to initiate a spike which depends on the exact position of the electrode, its polarity, and its current amplitude. Whether a spike can actually propagate along the cell is another question as blocking phenomena might prevent this^18,19^. In the investigated cases of this study, spikes were initiated mainly in the sodium band and the soma. Figure 4 shows the minimum spike threshold for a cathodic (negative) electrode above the soma of the GC. In this case the electrode initiates a spike within the soma because of the short distance of only 10 µm between electrode and soma surface. Moving the electrode a bit to the left side increases the shortest distance and consequently the threshold current, too. Within a range of several micrometers, spike initiation was observed within the soma but a larger horizontal shift causes the axon hillock to be closer to the electrode. Under the conditions of Fig. 4, there is a range of more than 10 µm where the electrode shift seems to be most effective in the hillock as it causes, according to the activating function (comp. Figure 2), hyperpolarization in the sodium band segment. During the first part of a supra-threshold cathodic stimulus sodium channels in the hillock open and axonal current flow causes depolarization in the sodium band leading to an AP peak in the band before the spike can develop in the hillock. Obviously, the sodium band plays a dominant role in spike initiation because of its high ion channel density. This is in accordance with published results^20^.

Figure 5a summarizes the sensitivity of the PC for a similar situation as presented for BC and GC in Fig. 4. In comparison with GC, the PC model includes a myelinated axon causing a zig-zag curve for cathodic stimulation. For cathode positions at the myelinated axon APs were initiated in the nearest node of Ranvier. Electrode positions above the dendrite need higher thresholds than above the axon, a consequence of sodium channel densities. A − 50 µA stimulus is just below threshold for the electrode position marked with a red+. A bit stronger stimulus (not shown) would cause a dendritic spike developing from the red line (Fig. 5b left upper case). When changing polarity (120 µA, Fig. 5b left lower case) the right side-wing of the activating function becomes prominent (comp, red line). Interestingly this first excitation in the dendrite does not initiate a spike there. However, this pre-excited dendritic region elicits a spike in the sodium band via intracellular current flow that back-propagates into the dendrite again. Figure 5 demonstrates that (i) for all investigated electrode positions APs can be elicited with both stimulus polarities, (ii) the spike initiation site is often close to the electrode position for cathodic stimulation, but shifted to the side-wings of the activating function for anodic stimulation, and (iii) there is a trend to elicit the spike in the sodium band via intracellular current flow. See^21^ for additional information. A similar variability in spike initiation is also found in experimental studies^19,22–24^.

**Figure 6:**
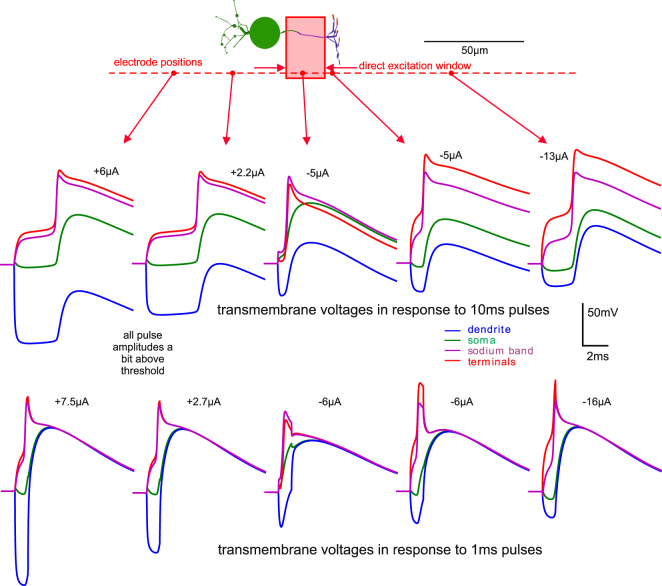
Transmembrane voltages evoked by anodic and cathodic extracellular stimulation. Extracellular sodium spike initiation in BC is frequently caused by strong depolarization in the terminals, the only exception is the electrode position directly above the sodium band. Note that in 4 of the 5 shown cases, the voltages in the terminals are larger than in the sodium band, both for 10 and 1 ms pulses. Only for electrode positions within the direct excitation window, marked as red rectangle, spikes are generated by depolarization of the sodium band membrane. Electrode distance is 20 µm from the neuron axis, resting membrane voltage is −70 mV.

The main differences between the BC and the other two model neurons are its small cell size and that the sodium ion channels are only found within the sodium band. Theoretically, a BC sodium spike should always be initiated in the sodium band as this is the only region where sodium ion channels are present at all. However, the results of the BC simulation show another situation: When the stimulating electrode is moved along the axon at a distance of 20 µm, there exists only a short region where the sodium spike is directly initiated within the band (compare transmembrane voltages right after stimulus start in Fig. 6). For all other positions, strong transmembrane voltages in the terminals are the driving elements to elicit the spike via intracellular (axonal) current flow. This is especially evident from the cathodic stimulation graphs shown in Fig. 6 right, where the membrane voltage in the terminals is distinctly larger than in the sodium band.

## Discussion

A physiological realistically generated BC sodium spike is processed in the following way (simulated but not shown): (i) Neurotransmitter released from photoreceptors causes a depolarization of the dendritic membrane voltage, (ii) ion current flow into dendrite and soma via T-type calcium channels causes insignificant signal amplification, (iii) the several hundred times higher density of sodium channels in the sodium band enables this BC to generate a spike which arrives with very short delay in the terminals (compare spikes of Figs. 3 and 6).

In the case of small synaptic input signals that are insufficient to be amplified by the sodium ion channels, the cell only generates graded potentials at the terminals. In such a subthreshold regime, the amount of neurotransmitter release is rather proportional to the dendritic membrane voltage as long as it is not influenced by the synapses of amacrine cells^4,11,25^. In contrast to other types of neurons, the calcium channels of the investigated BC support the initiation of sodium spikes, but, because of their low density, the generated ionic current by these channels is not high enough to initiate a dendritic calcium spike.

A gedankenexperiment underlines why no extracellular microelectrode position could be found in the dendritic region to imitate the natural spiking process mentioned in the paragraph before. Placing an anodic microelectrode in the two left positions of Fig. 6 is causing hyperpolarization of dendrites and soma, whereas the sodium band and the terminals are depolarized. Changing the electrode polarity to cathodic stimulation results in a situation comparable to natural spiking. Indeed, dendrite and soma become depolarized and an inward calcium current flow will be initiated. However, now the sodium band is hyperpolarized and, compared to the natural situation where it was assumed to be at rest, initiation of sodium channel opening would require a strong inner-axonal current flow to overcome the hyperpolarization. However, the limited calcium channel density in dendrites and soma is not sufficient enough to produce the required current in the investigated BC. A detailed discussion on dendritic spike initiation of the PC is found in ref.^21^.

Direct initiation of sodium spikes of the presented BC with an extracellular microelectrode is possible only for electrode positions within a small region, marked as direct excitation window in Fig. 6. This is a surprising result of this study. For other positions, spikes are only generated via antidromic current flow from the terminal region (Fig. 6). In such cases, there is a strong depolarization which may release a similar amount of neurotransmitter as a sodium spike itself. This result seems to be important for subretinal implants where placing tips of penetrating electrodes within a small spatial window close to a BC soma is problematic.

The best electrode position to elicit a sodium spike is close to the sodium band when cathodic pulses are applied. This general rule of thumb for non-myelinated sections of a neuron is of course valid for BCs, GCs, and PCs; it was also confirmed in all presented cases of this study. What seems to be an exception, is the minimum cathodic stimulation threshold of the GC case of Fig. 4. While one of the minima is just next to the sodium channel, there is a second minimum next to the soma. However, the electrode distance in the simulation was hold constantly 20 µm above the axon axis. Therefore, the second minimum is a consequence of the local high stimulation strength caused by the short distance of the electrode to the soma surface.

Another rule of thumb states that anodal thresholds of neurites are about 4 times larger than the cathodal ones. This is observed in different experiments (e.g.^21^) and mathematically described by the activating function concept^20,26,27^. Also here in this study, this rule holds roughly for GC as shown in Fig. 4 and for the PC (Fig. 5a). In contrast the BC again, here this rule fails as there are no direct sodium spikes found at all for anodal stimulation.

A last rule underlined by theory and experiment^28^ is the reversed recruitment order for electrical stimulation of motor neurons with the consequence that small axons are harder to stimulate than thicker ones. Thus, the much smaller diameter of BC vs. GC (Table 1) underlines why the model BC has essentially higher thresholds than the GC for cathodic stimulation (Fig. 4).

## Methods

The BC model is based on the reconstructed 3D morphology of a retinal DB4 bipolar cell from macaque and ion channel dynamic data^9^. The soma of the cell was replaced by a sphere with the same surface as the real cell’s soma. For all channels Hodgkin-Huxley type kinetics are used. Sodium channel Nav1.1 density distribution in the sodium band has a peak represented by g_Na_ = 1000 mS/cm² (Fig. 1). The kinetics of m,h and s Nav1.1 gates were defined by Vhalf = −27.2, −60, −60 mV with slopes z = 4.6, 7.7, and 5.4 mV respectively^29,30^. The T-type Cav3.1 is assumed to be of constant density (g_Ca_ = 2 mS/cm²) in soma, dendrite, and axon hillock compartments. Its m and h gates are defined by Vhalf = −57, −81 mV and slopes z = 6.2 and 4 mV respectively^31^. There are two types of potassium channels, a fast and a slow one. The fast type is located in sodium band with g_K_ = 2 mS/cm² while the slow potassium channels are located at soma, dendrite, and axon hillock with gk = 2.4 mS/cm². This conductance value was derived from the voltage clamp experiments in^9^. The kinetics of potassium channels are based on the original Hodgkin-Huxley potassium channel, but include a voltage offset and a tau rate which slows down the channel by the given factor. The fast potassium channel of the sodium band is shifted by 5 mV and has a tau rate of 5 (means 5 times slower than standard Hodgkin-Huxley potassium channel) while the slow channel has no voltage shift and tau rate of 30. L-type Cav1.4 and HCN1 channels are located in terminals with densities 10^−4^ and 10^−5^ mS/cm^2^ ^32^, respectively with kinetics used in^4,33^. Leak current conductance g_L_ = 0.033 mS/cm² and membrane capacitance C_m_ of 1 µF/cm² are taken from^9^. For all of the calculations temperature coefficients were used to simulate the dynamics at 31 °C^34^. For threshold detection a BC spike amplitude of 60 mV was used for Figs 4 and 5. Python 2.7 were used for modeling and graphs.

The GC model is based on traced data^35^ and simplifications as published in ref.^18^. A sketch of the geometry is included in the upper part of Fig. 4. The cell consists of a spherical soma (d = 20 µm), an axon hillock (40 µm, d = 3 µm), a sodium band (40 µm, d = 2 µm), a thin axon (720 µm, d = 1 µm), a vertical dendrite segment (10 µm, d = 4 µm), and two dendrite branches each (300 µm, d = 2 µm). The model of ref.^36^ at (22 °C) was used with fitted sodium channel conductivities corresponding to ref.^18^ and ^35^: g_Na_ values in mS/cm² in brackets are: dendrites (25), soma and hillock (80), sodium band (400), distal axon (70). Assuming constant ratios between single conductivities, all other conductivities (potassium, calcium, leakage) were determined based on the respective g_Na_ of each section and the ratios as given by ref.^36^. The model and a user interface were created in Python 2.7 using Neuron as modelling software.

The PC model is a simplification of a real cortical pyramidal cell^13,15,17,23^. It has a straight axis and consists of a single non-branching dendrite (500 µm, d = 5 µm), spherical soma (d = 20 µm), axon hillock (10 µm, d = 3.1 µm), AIS (50 µm, d = 1.22 µm), naked axon (unmyelinated, 200 µm, d = 1 µm), myelinated axon (500 µm, d = 1 µm), and unmyelinated terminal (50 µm, d = 1 µm). Assumptions for ion channel distribution and ion current computations are quite similar as in^15^: the same constant Nav1.2 channel density for dendrite and soma (g_Na_ = 8 mS/cm²), but 40 times higher sodium channel density in hillock and AIS with a change to the low threshold type Nav1.6 in the axon. For details see^21^. Matlab was used for simulations.

Intracellular resistivity was 0.1 kOhm.cm, retina resistivity 1 kOhm.cm^4,37^; both values were used for BC and GC. Because of the high neuron density in the retina averaged resistivity values up to 5 kOhm.cm are reported^38^. For PC 0.1 kOhm.cm and 0.3 kOhm.cm were used for intra- and extracellular resistivity, respectively.
